# Comparison of Periodic In-person ID Care to Daily Tele-ID Care at a Community Hospital

**DOI:** 10.1093/ofid/ofaf371

**Published:** 2025-06-24

**Authors:** Sowmya Nanjappa, Peter Volpe, Nupur Gupta, Sui Kwong Li, Christian Perez, Kate Gass, John W Mellors, Rima C Abdel-Massih

**Affiliations:** Division of Infectious Diseases, University of Pittsburgh Medical Center, Pittsburgh, PA, USA; Infectious Disease Connect, Inc., Pittsburgh, PA, USA; Division of Infectious Diseases, University of Pittsburgh Medical Center, Pittsburgh, PA, USA; Division of Infectious Diseases, University of Pittsburgh Medical Center, Pittsburgh, PA, USA; Infectious Disease Connect, Inc., Pittsburgh, PA, USA; Division of Infectious Diseases, University of Pittsburgh Medical Center, Pittsburgh, PA, USA; Infectious Disease Connect, Inc., Pittsburgh, PA, USA; Division of Infectious Diseases, University of Pittsburgh Medical Center, Pittsburgh, PA, USA; Infectious Disease Connect, Inc., Pittsburgh, PA, USA; Infectious Disease Connect, Inc., Pittsburgh, PA, USA; Division of Infectious Diseases, University of Pittsburgh Medical Center, Pittsburgh, PA, USA; Infectious Disease Connect, Inc., Pittsburgh, PA, USA; Division of Infectious Diseases, University of Pittsburgh Medical Center, Pittsburgh, PA, USA; Infectious Disease Connect, Inc., Pittsburgh, PA, USA

**Keywords:** community hospital, infectious diseases specialists, remote care, tele-ID, telemedicine

## Abstract

**Background:**

Access to infectious diseases (ID) specialists is absent in most US counties. Remote access to ID specialists via telemedicine is a potential alternative to in-person ID care, although comparative data are lacking. Armstrong Center for Medicine and Health, a 164-bed community hospital located in Armstrong County, Pennsylvania, that historically had periodic access to an in-person ID specialist every third business day established daily Tele-ID services in April 2020. We compared outcomes between periodic in-person ID care and daily Tele-ID care.

**Methods:**

We performed a retrospective chart review comparing the outcomes of 100 patients seen via periodic in-person ID care from January 2019 to November 2019 versus 100 patients seen via daily Tele-ID care provided through live audio-video telemedicine visits and electronic consults (e-consults) from May 2020 to August 2020.

**Results:**

The daily Tele-ID care group had significantly higher Charlson Comorbidity Index scores (5.3 vs 4.5, *P* = .047), shorter length of stay (7.5 vs 9.08 days, *P* = .003), were less likely to be discharged on intravenous antibiotics (34% vs 51%, *P* = .007), and had more frequent discharges on oral antibiotics (39% vs 23%, *P* = .014; Table 2). There were no significant differences in the rates of transfer to tertiary care facilities (13% vs 14%, *P* = .84) or in-hospital mortality (2% vs 2%). The 30-day readmission rate was higher for the daily Tele-ID care group (11% vs 1%, *P* < .01); however, only 1 readmission was ID-related (*Clostridioides difficile* infection). The diversity of ID diagnoses made between the periodic in-person ID care and daily Tele-ID care groups was similar, except for increased rates of tickborne illnesses diagnosed via daily Tele-ID care (Figure 1). There was greater utilization of daily Tele-ID care compared to periodic in-person ID care.

**Conclusions:**

A Tele-ID service with daily availability increased the utilization of ID care at a community hospital. Despite higher Charlson Comorbidity Index scores in Tele-ID patients, length of stay was shorter and discharge on intravenous antibiotics was less frequent with no statistically significant difference in ID-related readmissions or in-hospital mortality. These findings support daily Tele-ID care as a potential solution where access to ID expertise is limited.

## Teaser

Across the United States, timely access to ID specialistsis challenging for patients and providers. However, a daily inpatient Tele-ID servicecan not only mitigate access issues but ensures timely care leading to improved outcomeswhile keeping patients in their community.

During the height of the COVID-19 pandemic in 2020, Walensky et al demonstrated that almost 80% of US counties do not have a single infectious disease (ID) physician. This suggests that >200 million people living in predominantly rural areas of the United States have no access to ID specialists [1]. ID consultants play a vital role in coordinating multidisciplinary patient care. Evidence suggests that ID consultation decreases mortality for *Staphylococcus aureus* bacteremia, *Pseudomonas aeruginosa* bacteremia, multidrug-resistant organism infections, and candidemia [2–6]. Many community hospitals are unable to provide ID expertise needed to treat these severe infections due to geography limitation, costs, and a decreasing number in the ID workforce. To help mitigate these challenges, the Infectious Diseases Society of America has supported the appropriate use of telehealth technologies to provide effective ID subspecialty care to access-limited populations [7]. Although Tele-ID has expanded its presence within these underserved areas, there are limited studies suggesting how efficacious the care is [8]. Here, we report the outcomes between periodic in-person ID care and daily Tele-ID care at a community hospital.

## METHODS

University of Pittsburgh Medical Center (UPMC) has a renowned ID division providing in-person consultation at Pittsburgh-area hospitals. To meet the western Pennsylvania needs, the division established its first Tele-ID service in 2013 and has since continued to grow this model across the United States [9]. The Armstrong Center for Medicine and Health, a 164 licensed bed hospital located in Armstrong County, Pennsylvania, has had periodic access to an independent in-person ID specialist every third business day. Following their departure, a Tele-ID service was established at Armstrong Center for Medicine and Health by the UPMC ID division in April 2020.

We performed a retrospective chart review comparing the outcomes of successive 100 patients seen via periodic in-person ID care from January 2019 to November 2019 versus 100 subsequent patients evaluated via daily Tele-ID care from May 2020 to August 2020. April 2020 was excluded because it was the first month of establishing a new platform for an ID consultation service. We extracted race, gender, age, and calculated Charlson Comorbidity Index (CCI). Outcomes included total hospital length of stay (LOS), post-ID consult LOS, unique ID diagnosis, *Clostridioides difficile* infection, discharge on intravenous (IV) antibiotics or oral antibiotics, discharge disposition, in-hospital mortality, transfer to tertiary care facility, reasons for transfer, and readmission rates.

All statistical significance analyses were performed using the Shapiro-Wilk test for normality and using the Mann-Whitney *U* test, *t*-test, and 2-sample proportion tests for comparison. The study did not require institutional review board approval because it was approved by the UPMC quality improvement committee (Project ID 2046: Assessing the Quality and Outcomes of the Infectious Diseases Services Provided via telemedicine within the UPMC Healthcare System).

### Tele-ID Service Structure

Starting in April 2020, 1 full-time equivalent Tele-ID physician from a pool of 5 rotating ID physicians provided live audio-video visits, electronic consults (e-consults), and telephonic physician-to-physician consults. Live and asynchronous consults and follow-ups were available Monday–Friday from 8 Am to 5 Pm local time, with telephonic coverage being available 24/7, including weekends and holidays. The initial and follow-up live visits were performed with the assistance of a trained local tele-presenter registered nurse (RN). The RN was trained to use the telemedicine software program and perform a complete physical examination including the use of a Bluetooth-enabled stethoscope to allow auscultation. E-consults consisted of reviewing electronic medical records, discussion with local treatment teams, and charting appropriate documentation. The decision to perform live audio-video visits versus e-consults was at the discretion of the Tele-ID physician based on patient complexity and RN availability. Following hospital discharge, patients were followed by local community providers. No specific antimicrobial stewardship strategies were implemented in this Tele-ID model other than what the Tele ID physician provided in their consult recommendations.

## RESULTS

The Tele-ID service saw increased utilization when compared with the in-person ID care group: 100 consults were performed in a 4-month duration versus an 11-month duration in the periodic in-person ID care group. In the daily Tele-ID care group, there were a total of 73 live initial consults and 27 initial e-consults.

The mean age was 63.6 years in the periodic in-person ID care group versus a mean age of 67.8 years in the daily Tele-ID care group. One hundred percent of patients in the periodic in-person ID care group were Caucasian versus 98% of patients in the daily Tele-ID care group. Fifty-nine percent of patients in the periodic in-person ID care group were male versus 56% of patients in the daily Tele-ID care group. The mean CCI score was 4.5 in the periodic in-person ID care group versus 5.34 in the daily Tele-ID care group with an alpha risk of 0.05 and *P* value of .047 (Table 1).

**Table 1.** Patient Demographics of the Study Population

	In-person ID Service	Tele-ID Service	*P* Value
Total encounters	100	100	
Mean age (y)	63.59	67.84	.0553
Caucasian (%)	100	98	1.552
Male (%)	59	56	.668
Charlson Comorbidity Index score (mean)	4.5	5.3	.047

Abbreviation: ID, infectious diseases.

The total hospital LOS in the periodic in-person ID care group was a mean of 9.08 days versus a mean of 7.5 days in the daily Tele-ID care group with a significant *P* value of .003 (Table 2). Patients were discharged on antibiotics at an equivalent rate in both groups (74% in the periodic in-person ID care group vs 73% in the daily Tele-ID care group); however, more patients were discharged with IV antibiotics in the periodic in-person ID care group compared to the daily Tele-ID care group (51% vs 34%, *P* = .007). Additionally, more patients were discharged on oral antibiotics in the daily Tele-ID care group as compared to the periodic in-person ID care group (39% vs 23%, *P* = .014).

**Table 2.** Outcomes of the Study Population

	In-person Infectious Diseases Service	Tele-ID Service	*P* Value
Average LOS (d)	9.08	7.5	.003
Discharge on IV antibiotics (%)	51	34	.007
Discharge on PO antibiotics (%)	23	39	.014
Transfer to tertiary care center (%)	14	13	.84
In-house mortality (%)	2	2	-
30-d readmission (%)	1	11	.007

Abbreviations: ID, infectious diseases; IV, intravenous; LOS, length of stay; PO, oral.

There was insufficient power to determine a statistical difference in disposition to nursing facilities or hospice; however, there was no statistically significant difference in disposition to home between the 2 groups. Rates of transfer to tertiary care facilities were similar between the periodic in-person ID care group and daily Tele-ID care group (14% vs 13%, *P* = .84). In-hospital mortality was equivalent in both groups at 2%. Thirty-day readmission rate was higher in the daily Tele-ID care group compared to the periodic in-person ID care group (11% vs 1%, *P* = .007); however, further review noted only 1 of the 11 total readmissions in the daily Tele-ID care group was due to an infection-related adverse event (*C difficile* infection).

**Figure 1.**
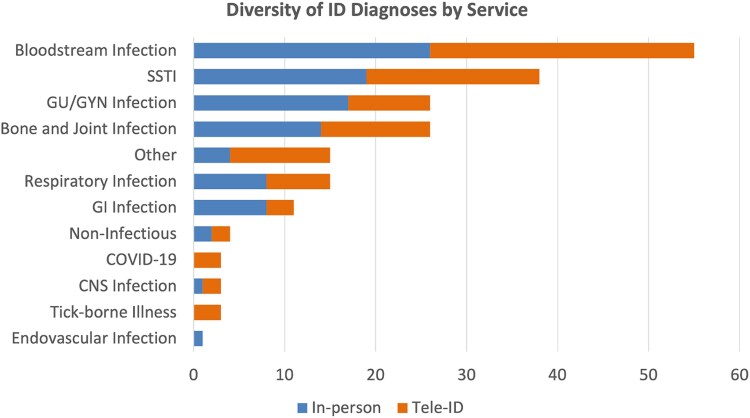
ID diagnosis of the study population. GI, gastrointestinal; GU/GYN, genitourinary/gynecology; ID, infectious diseases; SSTI, skin and soft tissue infection.

The diversity of ID diagnosis was similar between both groups including for bloodstream infections, skin and soft tissue infections, and genitourinary infections; however, there were higher rates of diagnosis of COVID-19 infection and tickborne illness in the daily Tele-ID care group given the timeframe of the study (Figure 1).

## CONCLUSIONS

This study supports Tele-ID efficacy at a community hospital as daily availability increased the utilization of ID care without sacrificing clinical outcomes. Despite higher CCI scores, LOS was significantly shorter and discharge on IV antibiotics was significantly less frequent in the daily Tele-ID group compared with the periodic in-person ID group. There was no difference in ID-related readmissions, in-hospital mortality, or transfer to a tertiary care center.

Although non–ID-related readmissions were higher in the Tele-ID group, this likely reflects the increased complexity of patients seen by this group. While transfers to tertiary care centers were equivalent in both groups, the numbers were low suggesting that access to ID care can allow patients to stay in their community hospital. None of the transfers in either group was related to the nonavailability of ID consultant expertise. The low in-hospital mortality rates were similar between the 2 groups despite Tele-ID seeing more complex patients. Discharge on fewer IV and more oral antibiotics was statistically significant in the Tele-ID group. This is important given the challenges associated with out-of-hospital infusions such as supply chain issues and staffing. Literature also supports a growing trend in ID toward more PO antibiotic use that are just as safe and effective as IV antibiotics for various conditions [10]. Last, the clinical ID diagnoses were similar in both groups suggesting a comparable diagnostic accuracy.

Limitations of the study include a smaller sample size and limited statistical power. The associations found may not be generalizable and uncertain if fewer consults were called in for in-person due to periodic availability. In our study, the university-based telemedicine provider group included 5 physicians versus a single in-person provider, and thus there may be individual clinical practice variations.

Furthermore, robust studies with larger sample sizes are needed for result validation and further insights. Overall, our study findings support remote Tele-ID care as a potential solution where access to ID expertise is limited.

## Notes

***Acknowledgments.*** The authors thank Keith Oelkers and Shannon Overly for all their assistance with statistical analysis.

***Author contributions.*** S.N., P.V., N.G., S.K.L., C.P., J.M., and R.A.M. contributed equally to this manuscript.

***Patient consent statement.*** Patient written consent not obtained does not apply to the paper. This study was exempt from institutional review board review.
